# Supplementary figures and images for: Comparing the performance of mScarlet-I, mRuby3, and mCherry as FRET acceptors for mNeonGreen
Source: PLoS One. 2020 Feb 5;15(2):e0219886. doi: 10.1371/journal.pone.0219886 (PMC7001971; doi:10.1371/journal.pone.0219886)

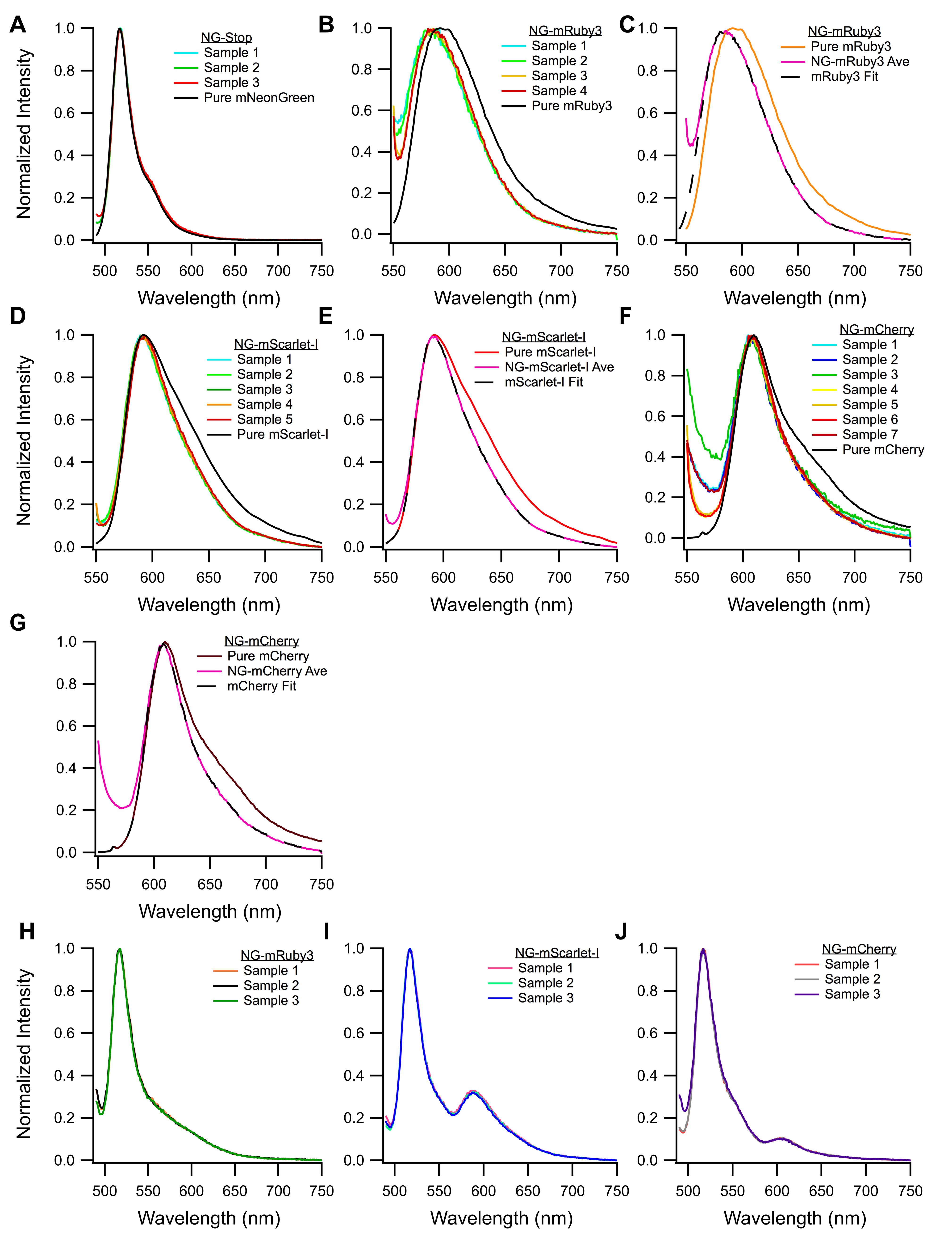

Supplement: S1 Fig — (A) Emission scans of several independent transfections of NG-Stop when excited at 470 nm overlaid with the reported emission of pure mNeonGreen. The average of these scans is shown in Fig 2B. Because of the consistency of NG-Stop with the pure mNeonGreen spectrum, the pure mNeonGreen spectrum was used as the donor spectrum for linear unmixing. Acceptor emission scans from several independent transfections of (B) NG-mRuby3, (D) NG-mScarlet-I, and (F) NG-mCherry achieved by exciting the acceptor directly using 530 and 540 nm light, overlaid with the reported pure spectrum for the red FP in each condition. The average of each condition is shown next to the reported pure spectrum for each acceptor is shown in (C), (E), and (G) respectively. Scan of both mScarlet-I and mCherry in the NG-mScarlet-I and NG-mCherry constructs faithfully replicated the upstroke and peak of purified mScarlet-I and mCherry, with the major difference between the observed and pure protein spectrum being a faster decay of the tail of the spectrum at high wavelengths. In contrast, the scans of mRuby3 in the NG-mRuby3 construct revealed an emission spectrum that was 6 nm shifted from what was reported for purified mRuby3. Due to the differences seen with each of the red acceptor proteins and varying levels of background, custom acceptor emission spectrums were created to serve as the acceptor emission for linear umixing shown in (C), (E), and (G) as the black dashed line. The raw traces used to determine the efficiency of (H) NG-mRuby3, (I) NG-mScarlet-I, and (J) NG-mCherry are shown, corresponding to the efficiency graph in Fig 2F. (TIF) [file pone.0219886.s001.tif]

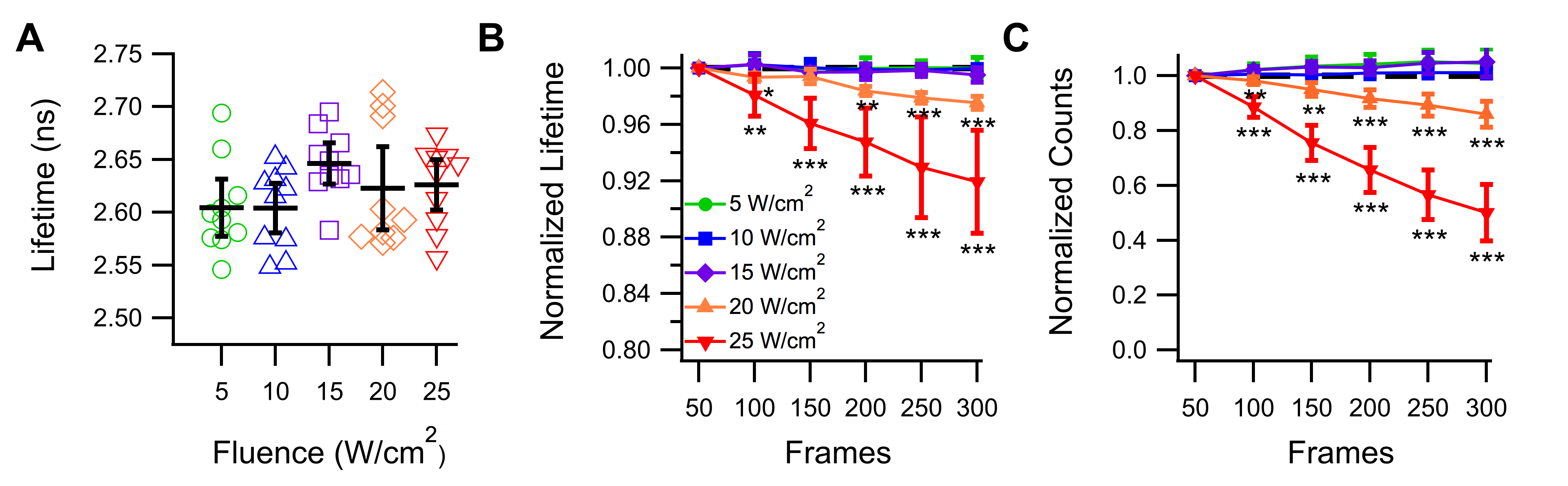

Supplement: S2 Fig — (A) Lifetime data collected from individual HEK293 cells expressing cytosolic EGFP at various laser powers up to 25 W/cm2 after 50 frames. Black bars indicate the average ± 95% confidence interval. (B) Lifetime and (C) intensity of samples taken over 300 frames at various laser powers. * = P < 0.05, ** = P< 0.005, and *** = P < 0.0005 compared to the frame matched 5W/cm2 dataset. N for each sample is as follows 5W/cm2: 10 cells, 10W/cm2: 10 cells, 15W/cm2: 10 cells, 20W/cm2: 9 cells, 25W/cm2: 10 cells. (TIF) [file pone.0219886.s002.tif]

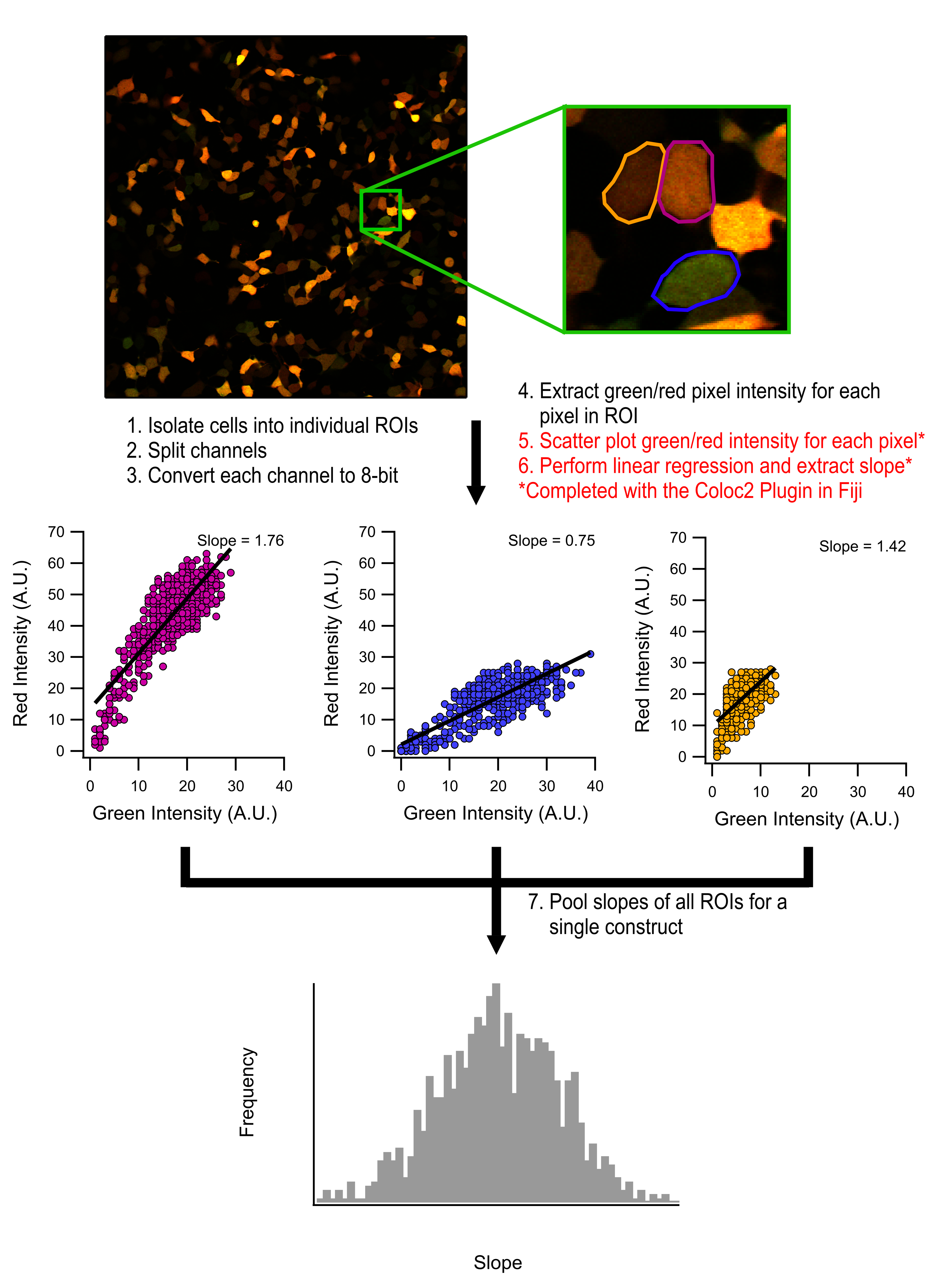

Supplement: S3 Fig — Example workflow demonstrating how confocal images were processed to create intensity slope histograms in Fig 5. (TIF) [file pone.0219886.s003.tif]

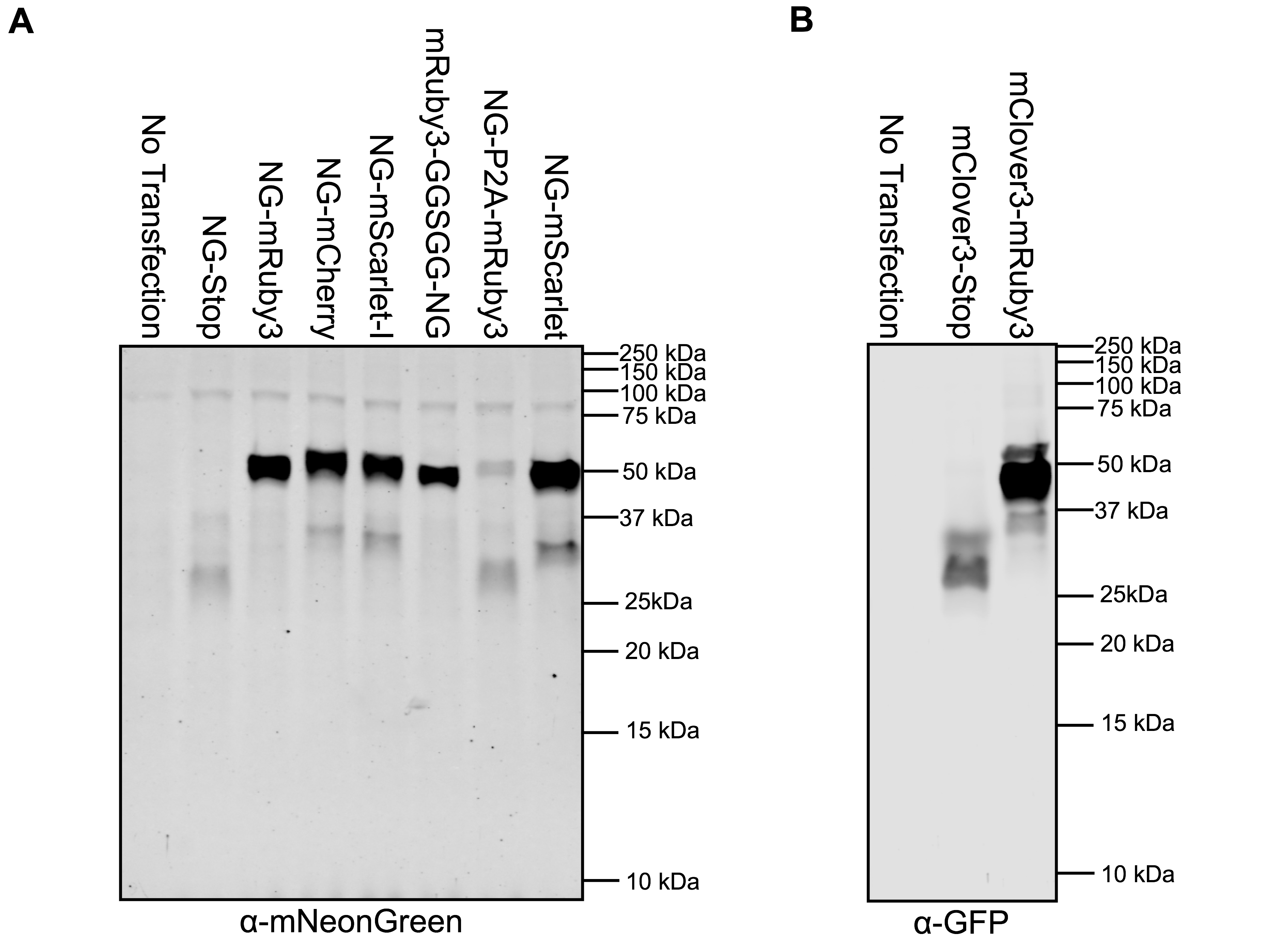

Supplement: S4 Fig — 10 μg of total protein derived from cell transiently transfected with the given construct one day post transfection was loaded into a 16% SDS-PAGE gel and mNeonGreen was visualized using an (A) anti-mNeonGreen antibody and (B) an anti-GFP (for the detection of mClover3 containg constructs). NG-Stop has presents a band near it’s predicted molecular weight of 27kDa. Each of the tandems except NG-P2A-mRuby3, show a bright band at the full predicted weight of the tandem constructs, 54kDA. Importantly, only the NG-Stop and NG-P2A-mRuby3 show bands corresponding to a monomeric mNeonGreen. NG-mCherry, NG-mScarlet-I, NG-mScarlet, and mClover3-mRuby3 show products in between the expected full tandem and the mNeonGreen monomer that are likely due to the hydrolysis of the red FPs backbone during cell lysis and subsequent protein denaturation that has been reported previously with DsRed like red FPs[38]. Note that the band that appears between 75 and 100 kDa in (A) is present in all lanes indicating that it is a non-specific target of the mNeonGreen antibody. (TIF) [file pone.0219886.s004.tif]

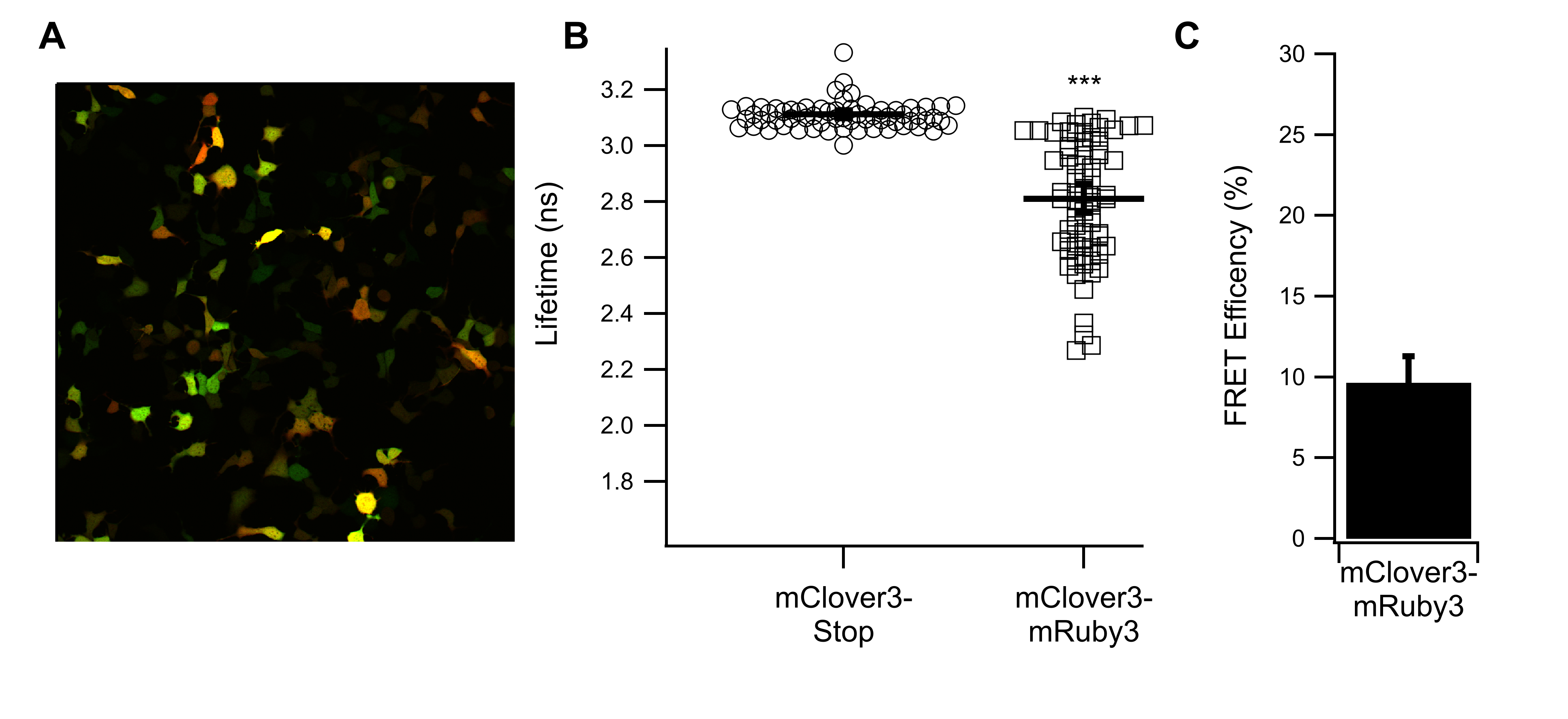

Supplement: S5 Fig — (A) Confocal merge image of HEK293 cells expressing mClover3-mRuby3 demonstrates that mClover3-mRuby3 also displays high expression heterogeneity, similar to what was seen with other mRuby3 constructs. (B) Lifetimes of HEK293 cells expressing mClover3-Stop (the donor only condition) and mClover3-mRuby3. Each symbol represents a measurement from a single cell. Black bars indicate the average ± 95% confidence interval. N for each sample is as follows, mClover3-Stop: 65 cells and mClover3-mRuby3: 71 cells. *** = P < 0.0005 compared to mClover3-Stop. (C) The average FRET efficiency of mClover3-mRuby3. (TIF) [file pone.0219886.s005.tif]

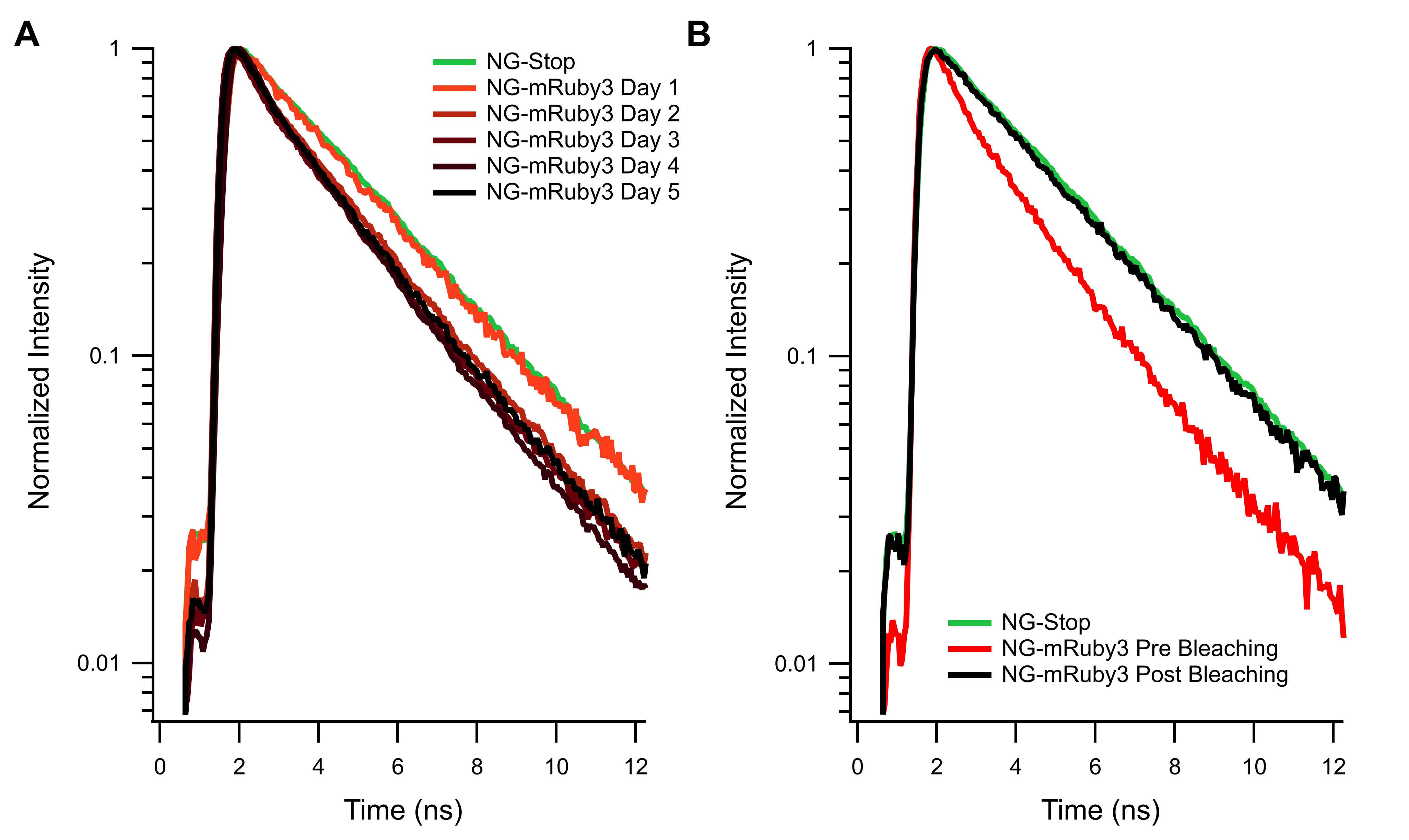

Supplement: S6 Fig — (A) Example fluorescence decay curves of a single cells expressing NG-mRuby3 2–5 days post transfection (DPT) representative of the average for each condition. The NG-Stop curve and NG-mRuby3 1 DPT curves from Fig 3C are also shown for reference. (B) Example fluorescence decay curves from a single cell expressing NG-mRuby3 5 DPT before and after acceptor photobleaching. The NG-Stop curve from Fig 3C is repeated here for reference. (TIF) [file pone.0219886.s006.tif]

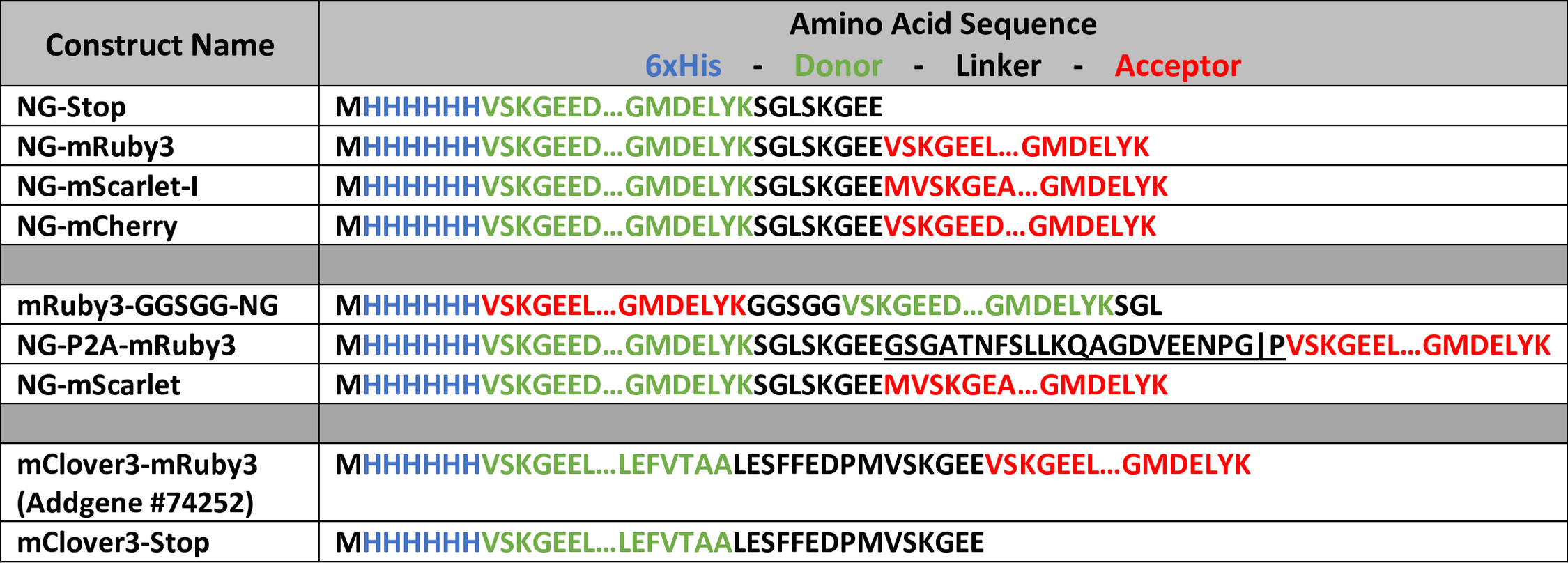

Supplement: S1 Table — The color-coded amino acid sequences for each construct are shown above. For NG-P2A-mRuby3, the cleavage site is found between the glycine and proline residues found immediately before the mRuby3 sequence and is indicated with a |. (TIF) [file pone.0219886.s008.tif]
