# Supplementary material for: Comparing the performance of mScarlet-I, mRuby3, and mCherry as FRET acceptors for mNeonGreen
Source: PLoS One. 2020 Feb 5;15(2):e0219886. doi: 10.1371/journal.pone.0219886 (PMC7001971; doi:10.1371/journal.pone.0219886)
Supplement: S1 Raw Images — Raw and unmodified immunoblot scans used in the construction of S4 Fig. (PDF) [file pone.0219886.s007.pdf]

S4_raw images

Figure derived from image: S3_Fig
(Split channel images only)

X indicates the lane is not depicted in the figure

Samples were processed as discribed in the Methods section of the maunscript. Samples were visualized using an Li-Cor Odyssey 3 Imaging System

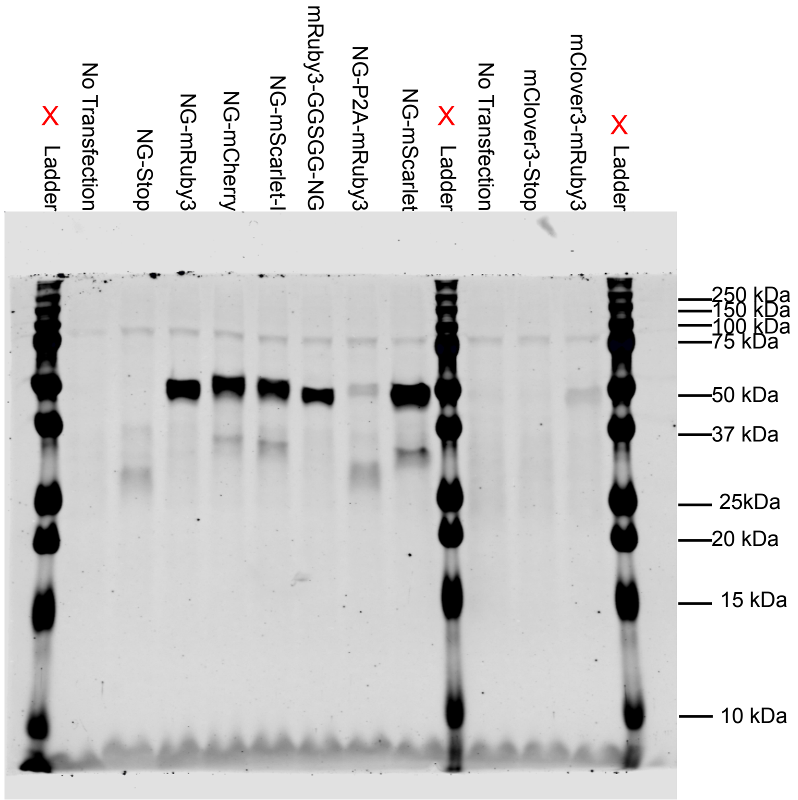

Primary: mouse α-mNeonGreen
Secondary: donkey α-mouse DyLight 680

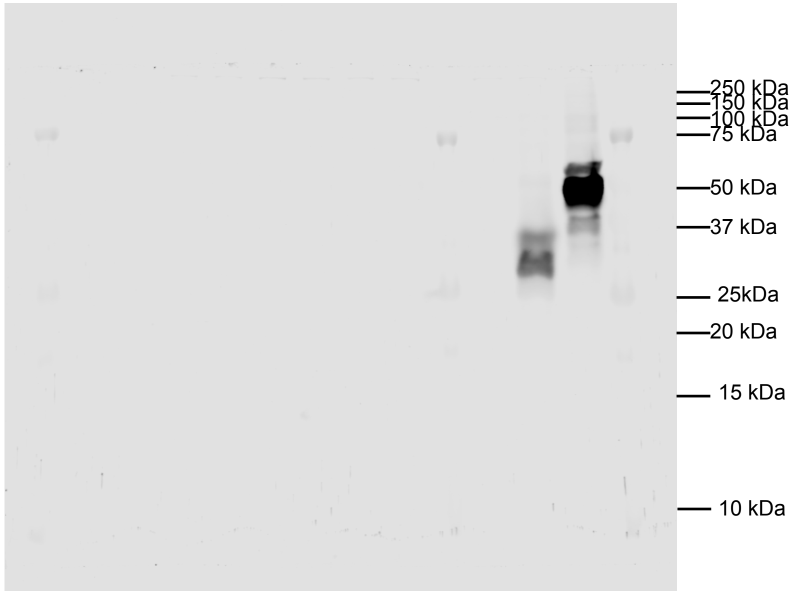

Primary: rabbit α-GFP
Scecondary: Goat α-rabbit DyLight 800

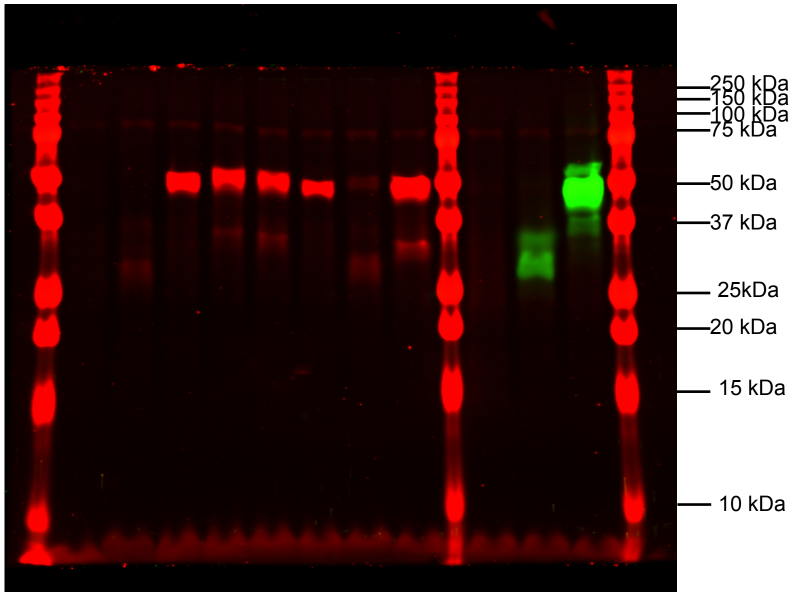

Merge
700 Channel: Red
800 Channel Green
