# Supplementary material for: Comparing the performance of mScarlet-I, mRuby3, and mCherry as FRET acceptors for mNeonGreen
Source: PLoS One. 2020 Feb 5;15(2):e0219886. doi: 10.1371/journal.pone.0219886 (PMC7001971; doi:10.1371/journal.pone.0219886)
Supplement: Attachment — Submitted filename: Response to the Reviewers.docx [file pone.0219886.s009.docx]

We would like to thank the editor and reviews for taking time to critically review our manuscript and for providing a valuable constructive critique of our work. We have read their comments carefully and have attempted to integrate their feedback into our revised manuscript to the best of our ability. This included the development and evaluation of several new constructs, as well as extensive text revisions within results and discussion sections. We will begin by addressing several comments that were common among more than one reviewer before addressing each reviewer’s remaining comments below.

General Comments

**Reviewers #1 and #3 both requested that we provide lifetime and photobleaching data for a more common FP under our 2-photon imaging system to act as a reference for mNeonGreen’s performance.**

We agree that repeating these experiments with an addition reference protein would be beneficial, so examined EGFP’s stability using the same methodology and report these findings in S2 Fig within the Supplemental Information as well as include a short discussion of the results with the results section.

**Reviewer #2 and #3 both expressed concern over the manuscript over-generalizing our results, stressing that we are testing our fluorescent proteins (FP) under a limited number of conditions which may not be representative of all possible FRET experiment using mNeonGreen (NG) and the various red FPs in our manuscript.**

We would like to thank the reviewers for pointing this out, as we certainly agree that our results may not be representative of all NG-red FP FRET. To address this, we made text edits to our manuscript where appropriate to stress that we developed and are testing a model system. Furthermore, we include a new paragraph at the end of the Discussion section to highlight the parts of our findings that may be more generalizable to other systems, while discussing how there may be unpredictable differences between our systems and others, stressing that it’s important to conduct further testing within an individual’s system of choice.

Reviewer #1

**1. The authors state that “...histograms of the slopes for individual cell expressing the NG-Cherry and NG-Scarlet constructs revealed a reasonable distribution and narrow spread of slopes (Fig 5C,D).” While it is true that both Cherry and Scarlet are narrower than Ruby, it does appear as though mCherry has a much narrower spread than mScarlet, and this would seem to represent a desirable property in a FRET acceptor. Given this, plus the fact that mCherry gave identical quenching to mScarlet in FLIM, suggests that (at least for FLIM) mCherry might actually be the better choice. This should be pointed out in the conclusion.**

Initially we were cautious of our interpretation of our slope data because it is unclear if mCherry’s narrower distribution was due to a higher level of expression/maturation efficiency or if its due to mCherry’s lower brightness, or both. That being said, the distribution of lifetimes we received for NG-mCherry was also narrower than that of NG-mScarlet-I, so we are willing to agree that mCherry did act as a more consistent acceptor and modified the text of the conclusion to include this.

**2. Most cells expressing NG-mRuby3 were observed to decrease their lifetime over the course of several days, suggesting that the low FRET efficiency observed 1 DPT was due to the slow maturation. Some cells were observed to still be exhibiting donor-only like lifetimes after 5 DPT. Perhaps a reasonable explanation for this result is cell-to-cell heterogeneity in terms of the rate of protein turnover? That is, cells that have faster turnover, and faster rate or protein synthesis, might be expected to never accumulate substantial amounts of the fully mature fusion protein, no matter how long they are cultured. One way to examine this might be to plot lifetime vs mNeonGreen fluorescence intensity. If this hypothesis is correct, brighter cells might be expected to have lower lifetimes due to slower turnover of the protein and accumulation to higher levels. In both the abstract and discussion, the authors emphasize that the reasons for the poor performance of mRuby3 are unclear. It does seem like, ultimately, it is the very slow maturation (and possibly efficiency of maturation and/or rate of protein turnover) that is the culprit. Based on the data in Table 1, that is the one characteristic in which mRuby3 is the outlier. I think that the authors could be bit more decisive in pinning the blame on this factor.**

We would like to thank the reviewer for the suggestion, as we do think it is likely that differences in protein turnover or stability from one cell to another causes the heterogeneity we observe with slow maturing acceptors (All mRuby3 constructs as well as a new mScarlet construct). Unfortunately, we are not able to conduct the suggested analysis due to our lifetime imaging methodology. While collecting lifetimes, it is important for us to keep the counts per second our detectors receive within a particular range to allow for accurate detection of lifetimes without overloading the detectors, which would cause artificial shorting of the lifetime (or damage to the detector in extreme situations). Because of this, we modulate the laser power used from field to field as necessary, which prevents us from making comparisons of cell intensity from one field to another. We did include a discussion of this idea within Discussion section though.

**3. The explanation that short linkers (line 487) are the reason why mCherry and mScarlet performed similarly does not make sense. The observed FRET efficiencies are all in the 20-30% range, meaning that the two fluorophores are at distances greater than the Forster radii. It is only when two fluorophores get very close that FRET efficiencies approach 100% and differences in Forster radii become irrelevant. I suspect that the two factors that really matter here are the time for protein maturation as well as the fraction of RFP proteins that make a fluorophore. I suspect that mCherry is particularly efficient at forming the chromophore, which is why it typically provides a bright signal in cells, even though its quantum yield and extinction coefficient are not as high as other proteins.**

We would like to thank the reviewer for pointing out the error in our logic and have replaced the relevant discussion section with one suggesting this may be due to mCherry’s maturation properties.

**4. I would like to suggest that the authors make it clear about the performance of these FPs for FRET (which only refers to donor quenching) and sensitized emission. For example, in the Conclusion the author’s state that “... but mScarlet-I outperformed mCherry in the intensiometric study due to its higher quantum yield.” It should be clear that the better performance is due to greater sensitized emission due to the higher quantum yield. Strictly speaking, the quantum yield of the acceptor is irrelevant to FRET (but critical for sensitized emission, of course).**

We have modified our conclusions to better clarify this point.

**- To someone who is not an expert in this field, the term 'better tissue penetrance’ in reference to green and red fluorophores. This should be reworded to make it clear that longer wavelengths of excitation and emission light are what better penetrate through tissue.**

We have added additional wording to clarify.

**- I think it is important to always include the ‘m’ and the ‘-I’ in the case of mScarlet-I, when referring to the FPs. Dropping these could cause some confusion for readers.**

We have modified the names of constructs throughout the paper (text and figures) to include the full names of the red FPs for clarity.

Reviewer #2

**1. The authors should be very clear in the caveat that these measurements were done using only one configuration (N-terminal mNeonGreen, C-terminal red FPs, only one linker used), and may not be generalizable to other constructs or sensors made using these FPs. The work was also done using only one donor and three acceptors, and thus is not generalizable to any other potential FRET pairs.**

Please see general comments above.

**2. The presence of a second, lower MW band on Western blots is not surprising when using red-emitting FPs with a DsRed-type chromophore, which all three acceptor FPs possess. This is due to hydrolysis of the red chromophore upon protein denaturation (the acylimine moiety is vulnerable to hydrolysis), and is generally not an indicator of proteolysis. The authors may wish to revise their discussion of this observation to reflect this.**

We would like to thank the review for pointing out that the acylimine moiety is vulnerable to hydrolysis. We have updated our discussion of the western blot results (which are now mostly contained within the S4 Figure caption) to specify that we were referring to proteolysis of the linker itself, and that extra bands between monomeric mNeonGreen/mClover3 and the dimer band are due to this process.

**3. Despite the fact that mRuby3 clearly does not perform well in this assay, it remains mysterious and concerning that the authors observed such extreme heterogeneity in their transfected cells even after several days of maturation time. From the description of the constructs, it seems possible that the same DNA sequence, encoding the amino acid sequence SKGEE, may appear three separate times in each construct, since all of the FPs being tested have "GFP ends," i.e. the first and last seven amino acids have been altered or appended to match those of GFP (MVSKGEE....GMDELYK). If it is indeed the case that this 15 base pair sequence is repeated three times in each construct, it is plausible that some recombination may occur after transfection. Ideally, the authors should fix this issue if it indeed exists.**

The reviewer is correct that this sequence does appear two or three times for all constructs. For clarity, we include a new supplemental table (S1 Table) to clearly disseminate the amino acid sequence of our constructs. As for recombination, we believe that it would be more likely to occur within bacteria while we were making or propagating the constructs, and we have assured that this is not happening through sanger sequencing of each construct before it was used in the mammalian cells. If recombination was to occur in the mammalian cells to a significant extent that resulted in mNeonGreen without a red protein acceptor, we should be able to detect that with western blotting, and we did not (S4 Fig). Given that data, and our confocal data which suggest that the major phenotype is mNeonGreen fluorescence without red FP fluorescence, we do not believe recombination within our HEK293 cells is a problem.

Reviewer #3

**1. The conclusions of this paper are written to apply to all uses of FRET but the example chosen of a tandem fusion between acceptor and donor is actually unlike any FRET reporter (it is more conformationally restricted than any FRET reporter would be). What about a real FRET reporter such as a single-chain kinase reporter (the prototype being AKAR)? And what about bimolecular reporters such as those used extensively by Ryohei Yasuda using FLIM?**

We hope that the information we disseminate in this manuscript will ultimately aid the development of new and exciting FRET systems of various kinds, including but not limited to biosensors. Although we are capable of testing these FPs outside of our model system, we do not believe there is a “standard” system that would enable fair comparison between the results we received with our model system and other systems, without testing each individual system. As reviewer #2 and #3 pointed out, the data we receive from our system is situational, but that assumption also applies to other systems as well. Given this, we do not feel like it will be an overly meaningful addition to develop and test a new set of reporters.

**2. Even within the confines of studying a donor-acceptor pair, only a single fusion protein (the mNeonGreen-mRuby3 protein) is being studied. It is premature to generalize or make predictions outside of this single protein.**

During the process of revising the manuscript, we developed several new constructs which are described below.

**3. Even within the even more restricted topic of whether mNeonGreen and mRuby3 can show high FRET coupling within a tandem fusion, then some simple troubleshooting is required at a minimum. Whereas previous studies used mClover3 and mRuby3 with good results, this study has swapped mClover3 for mNeonGreen. The new fusion protein may very well not allow folding of mRuby3 as well as previous fusion proteins.
a. For example, the mNeonGreen polypeptide may interfere with the mRuby3 polypeptide during protein folding; this might be the case either due to slower folding of mNeonGreen or specific peptide sequences within prefolded mNeonGreen binding to sequences of prefolded mRuby3. It is well known that domains of proteins can interfere with each other during folding, and natural proteins have evolved mechanisms such as translational pauses using rare codons and stiff linkers to allow domains to fold more independently.
b. Another reason for poor folding could be in the linkers themselves. It is will known that the sequence of linkers can greatly affect the folding of proteins attached to the linkers, e.g. George and Heringa 2002, PMID 12538905, or Chen et al 2014, PMID 23026637. Proline-rich and glycine-rich linkers are less likely to themselves interfere with the folding of an attached domain. Based on the authors’ description of their fusion protein, the linker between folded elements of mNeonGreen and mRuby3 has the sequence VMGMDELYKVSKEE, where the VMGMDELYK is brought in from mNeonGreen. This sequence contains a large proportion of hydrophobic and charged residues, which could interact with mRuby3 sequences to intefere with folding, and a scarcity of glycine or proline residues.**

We thank the reviewer for this discussion, and we agree that simply because our model system worked well for mScarlet-I and mCherry does not mean it was necessarily a good system to evaluate mRuby3. The study that originally published mRuby3 (*Bajar, et al (2016)*; ref #10) evaluated its performance as an acceptor for EGPF, mClover3, and mNeonGreen, and reported excellent results with each (35%, 45%, and 48% FRET efficiencies respectively). In that study they removed the “GFP ends” (GITHGMDELYK for EGFP and mClover3) or (GMDELYK for mNeonGreen) from the donors and fused them to mRuby3 (amino acids 3-233) with the following linker sequence: LESFFEDPMVSKGEE resulting in an effective linker of about 12 amino acids. Given this, we believe it’s is unlikely that an mNeonGreen which is 17 amino acids away would interfere with mRuby3 more than one that is approximately 12 amino acids away. Nonetheless, we developed two new tandems containing mRuby3: a construct with mNeonGreen fused to mRuby3’s c-terminus through GGSGG linker, and a construct in which a P2A self-splicing peptide was inserted between the mNeonGreen and mRuby3. Additionally, we tested a slow maturing variant of mScarlet-I, mScarlet. The data pertaining to these constructs are found in the new Fig 6, and lead us to conclude that factors intrinsic to mRuby3 are the source of its deficiencies as an acceptor, particularly its slow maturation. As for the composition of what we consider our “17 amino acid linker” in our first tandems (NG-mRuby3 and others), we chose to maintain the “GFP ends” of the proteins because we feel these are less frequently removed then they are kept.

**4. The authors chose to test only mNeonGreen as a donor. However it is not that much brighter than mClover3 (QY of 80% vs 78%), and the mRuby3 used here was actually taken out of the mClover3-mRuby3 fusion which does show high FRET. It would seem that the mClover3-mRuby3 should be used as a positive control for FRET (the authors could just use the existing construct they obtained from Addgene).**

We tested the mClover3-mRuby3 performance and report the data in S5 Fig, as well as a description of the results in the Results section. In summary, our data from this construct was more consistent our mNeonGreen donor data than what was previously reported for mClover3 as a donor (*Bajar, et al (2016)*; ref #10).

**5. Likewise the authors make no comparisons between mNeonGreen and other GFPs in fluorescence lifetime or brightness or photostability in their FLIM system. It was reported that mNeonGreen is as photostable as EGFP and more photostable than mClover3 in one-photon illumination, but relative photostability in two-photon illumination is unknown. The authors have already done the characterization for mNeonGreen, so it would be useful to characterize EGFP or mClover3 for comparison.**

Please see general comments above.
