# Supplementary material for: Comparing the performance of mScarlet-I, mRuby3, and mCherry as FRET acceptors for mNeonGreen
Source: PLoS One. 2020 Feb 5;15(2):e0219886. doi: 10.1371/journal.pone.0219886 (PMC7001971; doi:10.1371/journal.pone.0219886)
Supplement: Attachment — Submitted filename: Reponse to Reviewers_Revision.docx [file pone.0219886.s010.docx]

We again thank the editor and reviewers for their time, comments and thoughts regarding our manuscript. We have corrected typographical errors and ensured all figure references are to the correct figure, and that all references are consistently formatted to PLOS standards. The remaining points are addressed below.

**Reviewer #1: I would have liked to see the lifetime data for EGFP (Fig S2) with the data in Fig. 3ABC.**

The overall goal of our work is compare various red acceptors. The specific goal of Figure 3 is to establish that mNG is a good donor for the 2-photon FLIM experiments with which we will compare various red acceptors, not that mNG is superior to other specific donors such as EGFP. Therefore, we believe the supplemental data is an appropriate place for our EGFP data as more expert or multi-photon familiar readers can refer to that section for the comparison of donors while the general reader interested in acceptor behavior can move to the next section. We would like to point out that we took care to design Figure S2 to permit ease of comparison of mNG in Figure 3 and EGFP in S2 (keeping the scaling in the case of panel A and the axes for panels C and D that same, as well as the color coding and symbols used, and significance indicators) despite the data being in two separate places in the manuscript.

**Reviewer #3: I only suggest that they now add mClover3 and mScarlet to Table 1 ("Properties of the fluorescent proteins used in this study")**

We have added mClover3 and mScarlet to Table 1.

**In addition, the slightly higher lifetime of mClover3 vs mNeonGreen would suggest it should be an equivalent (or maybe slightly better) FRET donor for mScarlet-I. The discussion states "mNeonGreen is an ideal donor for Green/Red FRET experiments. Its yellow shifted excitation and emission spectrum allow for a high degree of overlap with red FPs while also being capable of being excited with lower energy blue light than cyan or more blue shifted green FPs." It might be appropriate to include mClover3 in this statement as well.**

We agree with Reviewer #3 that mClover3’s published properties would also suggest it would also make a suitable donor, similar to mNeonGreen. Unfortunately for the purposes of the manuscript, we did not extensively test mClover3 ourselves, and are therefore not comfortable making conclusions regarding how it may act as a donor and in Green/Red FRET experiments.
